# Multitrophic Interactions Between Arbuscular Mycorrhizal Fungi, Foliar Endophytic Fungi and Aphids

**DOI:** 10.1007/s00248-021-01937-y

**Published:** 2021-12-13

**Authors:** Nadia Ab Razak, Alan C. Gange

**Affiliations:** Department of Biological Sciences, Royal Holloway University of London, Egham, TW20 0EX Surrey UK

**Keywords:** Arbuscular mycorrhiza, Endophyte, *Impatiens glandulifera*, Inoculant, Insect

## Abstract

Almost all living plants can be simultaneously colonised by arbuscular mycorrhizal fungi in the roots and endophytes in the shoots, while also being attacked by insect herbivores. However, to date, no study has ever examined the multitrophic interactions between these two different fungal groups and insects on any species of forb. Here, we examined the effects of two commercial species mixtures of arbuscular mycorrhizal fungi (AMF) and two foliar endophytes (*Colletotrichum acutatum* and *Cladosporium oxysporum*) on the growth of an invasive weed, *Impatiens glandulifera*, and the aphids that attack it. AMF reduced plant biomass, which was most evident when *C. oxysporum* was inoculated. Mycorrhizal fungi had few effects on aphids, and these depended on the identity of the endophytes present. Meanwhile, endophytes tended to increase aphid numbers, but this depended on the identity of the AMF inoculum. Throughout, there were differences in the responses of the plant to the two mycorrhizal mixtures, demonstrating clear AMF specificity in this plant. These specific effects were also strongly affected by the endophytes, with a greater number of interactions found between the AMF and endophytes than between the endophytes themselves. In particular, AMF reduced infection levels by the endophytes, while some endophyte inoculations reduced mycorrhizal colonisation. We suggest that both AMF and endophytes could play an important part in future biological control programmes of weeds, but further multitrophic experiments are required to unravel the complexity of interactions between spatially separated parts of the plant microbiome.

## Introduction

Ever since the assertion that plants are not discrete entities, but instead harbour a considerable diversity of fungi within their tissues [1], ecologists have studied the pairwise interactions between these fungi and their hosts. However, the microbial community within plants, collectively known as the microbiome, is complex and contains an array of bacterial species, along with beneficial fungal symbionts in the roots (e.g. mycorrhizal fungi or dark septate endophytes), and other fungi in the shoots (e.g. endophytes, pathogens and other species that appear to be benign) [2, 3]. Despite various reviews lamenting the lack of multitrophic experiments involving the interactions between plants, their associated fungi and other trophic levels such as herbivores [4, 5], there is still a notable lack of studies that go beyond pairwise combinations to involve multiple microbial and/or herbivore species.

With the exception of plant pathogens, the most well-studied parts of the microbiome are Clavicipitaceous endophytes in grasses and the arbuscular mycorrhizal fungi (AMF) [4]. Pairwise interactions between these groups have often focused on the Poaceae, due to the agronomic and economic importance of this group. These studies have reported a wide variety of outcomes across many grass hosts, in which the fungi affect each other positively or negatively, with differing consequences for the host plant [6–8]. Such context-dependent results can be influenced by variation in abiotic factors, as well as the identity of the fungi and the plants concerned [9]. However, these interactions are important, as they propagate up food chains to affect insect herbivores. For example, colonisation of *Lolium perenne* by AMF tended to reduce the antagonistic effects of foliar fungal endophyte infection on larvae of the generalist moth *Phlogophora meticulosa* [10].

In contrast to grasses, microbial ecology studies in forbs (i.e. herbaceous plants that are not grasses, sedges or rushes) tend to be dominated by those involving AMF [4]. This is not surprising, given that up to 80% of forbs form an arbuscular mycorrhiza, with many important crop plants showing growth benefits when colonised by these fungi [11]. What is more surprising is that forbs also host a huge diversity of endophytic fungi in their roots and shoots [12, 13], yet nothing is known about the interactions of these fungi with their hosts, AMF and insects. These unspecialised endophytes in forbs comprise saprotrophs, latent pathogens, pathogens of other hosts (recently termed ‘schizotrophism’ [14]) and entomopathogenic fungi [12, 15]. Although the occurrence of these fungi in foliar tissues is often localised [16], they form a dynamic community, increasing plant resistance to insect herbivores and pathogens [17, 18], while having positive or negative effects on AMF, but often being antagonistic towards each other [19–21]. These endophytes therefore seem to be mainly beneficial to their hosts [15].

Arbuscular mycorrhizal fungi are also largely beneficial to their hosts, by increasing the nutrient supply and enhancing host plant resistance to some pests and pathogens [22]. However, it is widely recognised that ecological specificity exists in the mycorrhizal symbiosis [23], wherein the outcome of the interaction between fungi and the host is dependent on the identity of each, soil and environmental conditions, and the presence of other organisms, such as herbivores [24]. In certain conditions, AMF can lead to growth depressions in plants [25] while they frequently increase the growth and reproduction of sucking insect pests, such as aphids [26, 27]. From the commercial point of view, such detrimental effects on plants would not be a desirable feature of the mycorrhizal symbiosis, but one potential application occurs with the biological control of weeds [28]. Many annual weed species seem to be facultatively mycorrhizal at best and can show decreased growth, either as a direct effect of mycorrhizal colonisation, or from increased competition, due to neighbouring plants responding strongly to the presence of the fungi [28].

One invasive annual weed that seems to derive little benefit from AMF is Himalayan balsam, *Impatiens glandulifera*. Introduced from northern India and Pakistan in 1839, this plant is now regarded as one of the most invasive weeds in the UK and throughout Europe [29]. Studies have shown that the plant is antagonised by AM fungi in the introduced range and that it is able to reduce populations of indigenous fungi in the soil to its own advantage but to the detriment of native plant species [30–32]. This plant has been the subject of a biological control programme in Great Britain, where the host-specific rust fungus *Puccinia komarovii* var*. glanduliferae* was first released in 2014 [33]. Success of the pathogen has been patchy, due to variable biotype resistance in the weed and the presence of endophyte fungi, some of which are antagonistic towards the rust [34, 35].

Invasive species represent important ‘model systems’ in which to investigate plant–microbe-insect interactions, where there is a continuous search for control methods not involving chemicals or manual labour [36]. Here, we report on a study designed to investigate whether AMF and two endophyte fungi (*Colletotrichum acutatum* and *Cladosporium oxysporum*) interact to affect the growth of this weed and whether fungal presence has any effect on populations of two aphid species that naturally attack balsam, *Aphis fabae* and *Myzus ornatus*. This was part of a larger project examining the factors affecting, and consequences of, biological control of this weed [37]. As *I. glandulifera* seems to be antagonised by AMF species that do not occur in its native range, we hypothesise that using commercial mixed-species inoculants will reduce its growth. If so, this might then be exploited as a further biological control method for this weed. Our second hypothesis is that adding AMF will make the plant more susceptible to aphids, which could be problematic, as both aphids are pest species in the UK. However, as endophytes can be antagonistic to aphids, our third hypothesis is that endophyte presence might mitigate any mycorrhizal effect but that this might be further influenced by interactions amongst the endophytes themselves [17, 19, 26, 31]. To our knowledge, this is the first study to examine the interactions between various combinations of AM fungi, endophytes and insects in a non-grass host plant.

## Materials and Methods

### Plant and Fungal Material

Ripe *I. glandulifera* seeds were collected from wild populations at Harmondsworth Moor, Middlesex, UK (51.48°N, − 0.48°W) in autumn and stored overwinter at 4 °C for 6 months to break dormancy. Seeds were surface-sterilised and germinated on moist filter paper in sterilised Petri dishes at 4 °C.

In previous studies [34, 38], the two commonest endophytes isolated from *I. glandulifera* were *Colletotrichum acutatum* and *Cladosporium oxysporum*. The material was subjected to a molecular analysis for identification (described in full in ref. [34]) and deposited in GenBank with accession numbers MH428675 and MH428677, respectively, as well as the CABI culture collection (accession numbers IMI505519 and IMI505553, respectively). The material used here was from the same cultures studied by Currie et al. [34].

Two commercial mycorrhizal inoculants were used, provided by PlantWorks (Sittingbourne, Kent, UK) and Symbio (Wormley, Surrey, UK). The former (marketed as ‘Rootgrow’) contained *Claroideoglomus claroideum*, *Funneliformis geosporus*, *F. mosseae*, *Glomus microaggregatum* and *Rhizophagus irregularis*, while the latter (marketed as ‘Granular mycorrhizae’) contained *Claroideoglomus etunicatum*, *Funneliformis mosseae*, *Gigaspora margarita*, *Glomus deserticola*, *Gl. monosporus*, *Rhizophagus aggregatus*, *R. clarum* and *R. irregularis*. Hereafter, we refer to these two inoculants as ‘PlantWorks’ and ‘Symbio’ respectively.

### Experimental Design and Measurements

There were eight treatments for each mycorrhizal inoculant, consisting of with and without AMF, with and without *C. acutatum* infection and with and without *C. oxysporum*, with five replicates of each. One germinated seedling was planted into a 2-L pot, lined with a 38-μm membrane and containing John Innes number 3 compost (Westland Horticulture, Huntingdon). At planting time, 15 g of PlantWorks or 2 g of Symbio inoculum was added to the planting hole, following the recommended rates for horticultural crops. As the inocula differed in mycorrhizal species and carrier material, we sterilised each at 120 °C in an autoclave for 30 min. Thus, there was a different control of sterilised material for each inoculum type and so the two parts of the experiment were analysed separately (see Statistical Analysis, below). In addition, each control received a 15-mL microbial filtrate of live inoculum, in sterile water, passed through a 38-μm membrane, to remove all mycorrhizal propagules [39]. Inoculation with endophytes was identical to that by Currie et al. [34], when plants were at the three-leaf whorl stage, they were sprayed with an inoculum containing approximately 1.5 × 10^5^ spores mL^−1^ of *C. acutatum* and/or *C. oxysporum* in 0.05% Tween 80. Leaves were sprayed on the abaxial surface and control plants received equal volumes of 0.05% Tween 80 only.

Pots were sunk into field soil and arranged in a randomised block design. Each pot was rotated weekly, to prevent the ingress of AMF hyphae from the surrounding soil [40] and given 250 mL water daily. Plants were grown for 11 weeks, when flower buds had formed. Harvesting then took place, as the terms of the license (from the Animal and Plant Health Agency) for growing this invasive species do not permit flowering and escape of seeds into the wild.

Plants became naturally colonised by two species of aphid, *A. fabae* and *M. ornatus*, and total numbers of each species on each plant were counted prior to vegetation removal. Plant height and fresh shoot biomass were recorded, and a sub-sample of roots and leaves was taken for quantification of mycorrhizal colonisation and endophyte infection.

Roots were stained with the ink and vinegar method [41] involving immersion in 10% KOH at 80 °C for 25 min, thorough rinsing in water and subsequent immersion in staining solution (84.4:15:0.6, distilled water: 1% hydrochloric acid: Quink blue ink) for 30 min. Percent root length colonised by arbuscules was quantified with the cross-hair eye piece method [42], at × 200 magnification, with 100 views recorded for each slide of root pieces.

To examine infection levels by the inoculated endophytes, as well as infection by other species (background infection), five intact (i.e. showing no signs of herbivore attack) and asymptomatic (showing no signs of disease) leaves were randomly picked from each plant. Endophyte fungi were isolated from three 6-mm discs taken from each leaf, in an identical manner to that described by Currie et al. [34]. Briefly, surface-sterilised discs were plated onto potato dextrose agar (PDA) and individual colonies subcultured onto potato carrot agar (PCA). Sporulating cultures on PCA were identified by Dr B. C. Sutton, while sterile cultures were subjected to molecular identification, as described in Currie et al. [34].

### Statistical Analysis

Plant height and fresh biomass data were tested for normality and plots of residuals were examined. Biomass data were subjected to the logarithmic transformation, prior to analysis. As described above, the two parts of the experiment, involving the two mycorrhizal inocula, were analysed separately. Effects of mycorrhizal addition and that of the two endophytes were examined with three-factor analysis of variance. Aphid numbers and the number of background endophytes (i.e. non-inoculated species) per plant were analysed with a Poisson generalised linear model structure, using a log link function, having checked for overdispersion and employing mycorrhizal addition, and the two endophytes as main effects. Differences in percent root length colonised by mycorrhizal fungi were examined with analysis of variance, following the logit transformation [43], employing each endophyte as the main effect. The isolation frequency of each fungal endophyte species was calculated by dividing the total number of isolations (individual colonies) of each species in a plant by the total number of all fungal isolations for that plant [19]. Differences in isolation frequency were examined using a generalised linear model with a binomial error structure, after checking for overdispersion, and employing mycorrhizal addition and the two inoculated endophytes as main effects. All analyses were performed in R 4.0.2 [44].

## Results

All significant terms (main effects and interactions) are described below, and summaries of all statistical results are presented in Tables 1 and 2.

**Table 1:** Summary of the significant effects of, and interactions between PlantWorks inoculum (AM), inoculation with *Colletotrichum acutatum* (C.a.) and *Cladosporium oxysporum* (C.o.) on height and shoot biomass of *I. glandulifera*, total numbers of the aphids *Aphis fabae* and *Myzus ornatus*, arbuscular mycorrhizal colonisation (AMF) and isolation frequency of *C. acutatum* and *C. oxysporum*. For the main effects, ↑ represents an increase in the parameter, while ↓ represents a decrease. Significance is indicated by * *P* < 0.05, ** *P* < 0.01, *** *P* < 0.001

	Height	Biomass	*A. fabae*	*M. ornatus*	AMF	*C. acutatum*	*C. oxysporum*
AM	-	*↓	**↑	-		*↓	-
C.a	-	-	-	**↑	***↓	**↑	-
C.o	***↓	*↓	-	**↑	-	-	*↑
AM x C.a	-	-	-	-	-	-	-
AM x C.o	-	-	** AM effect only with C.o	* AM negates effect of C.o	-	-	* AM negates effect of C.o
Ca. x C.o	-	** C.a. negates effect of C.o	-	-	*** C.o. negates effect of C.a	-	-
AM x C.a. x C.o	* C.a. negates effect of AM and C.o	-	-	-	-	-	-

**Table 2:** Summary of the significant effects of, and interactions between Symbio inoculum (AM), inoculation with *Colletotrichum acutatum* (C.a.) and *Cladosporium oxysporum* (C.o.) on height and shoot biomass of *I. glandulifera*, total numbers of the aphids *Aphis fabae* and *Myzus ornatus*, arbuscular mycorrhizal colonisation (AMF) and isolation frequency of *C. acutatum* and *C. oxysporum*. For the main effects, ↑ represents an increase in the parameter, while ↓ represents a decrease. Significance is indicated by * *P* < 0.05, ** *P* < 0.01, *** *P* < 0.001

	Height	Biomass	*A. fabae*	*M. ornatus*	AMF	*C. acutatum*	*C. oxysporum*
AM	-	**↓	-	-		-	-
C.a	-	-	-	-	-	***↑	-
C.o	***↓	***↓	-	-	**↓	-	**↑
AM x C.a	-	-	-	-	-	*AM enhances effect of C.a	-
AM x C.o	-	-	-	-	-	**C.o. negates effect of AM	* AM negates effect of C.o
Ca. x C.o	-	-	-	-	-	-	-
AM x C.a. x C.o	-	-	* AM effect only with no C.a. & C.o	-	-	-	-

### Plant Growth Attributes

**Fig. 1:**
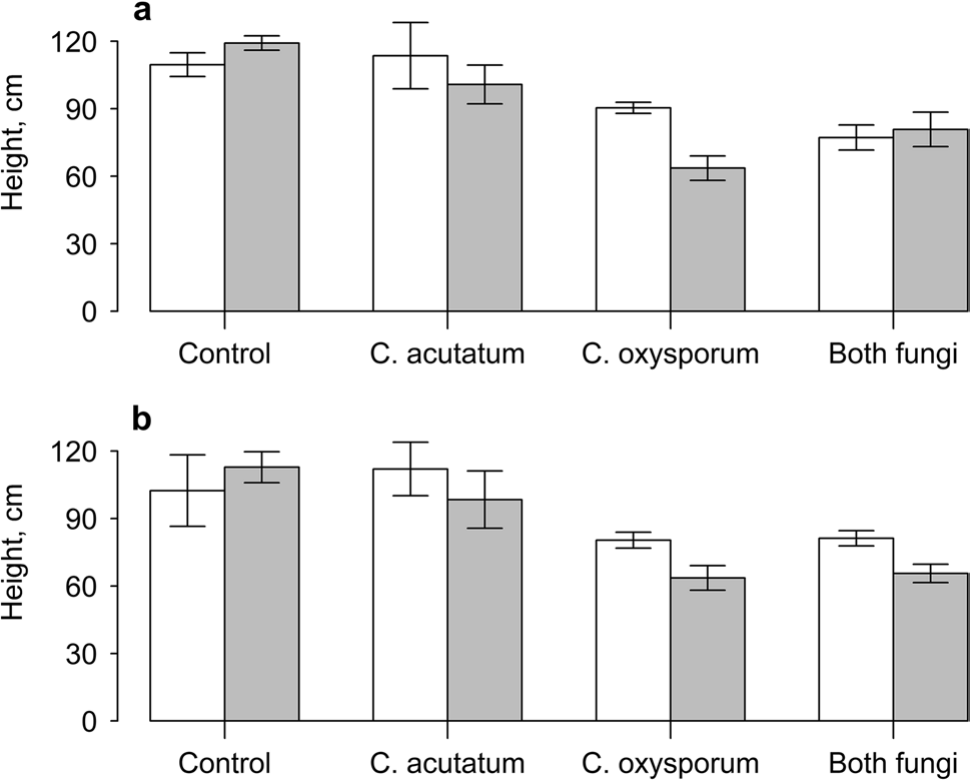
Height of Himalayan balsam plants grown with addition of PlantWorks mycorrhizal inoculum (**a**) or Symbio mycorrhizal inoculum (**b**). White bars indicate sterilised inoculum, grey bars indicate live mycorrhizal fungi. Plants were also inoculated with no endophytes (control), *Colletotrichum acutatum*, *Cladosporium oxysporum* or both endophyte fungi. Vertical bars represent ± one standard error

Overall, addition of PlantWorks mycorrhizal inoculum had no effect on plant height; however, plants infected with *C. oxysporum* in this part of the experiment were shorter (main effect: *F*_1,32_ = 38.21, *P* < 0.001, Fig. 1a). When this endophyte was present, mycorrhizal fungi appeared to reduce height still further, producing the shortest plants. However, this effect was not seen if *C. acutatum* was present also, leading to a three-way interaction between the fungi (*F*_1,32_ = 6.64, *P* < 0.05). An identical result was found with the Symbio inoculum, wherein *C. oxysporum* reduced plant height (main effect: *F*_1,32_ = 26.93, *P* < 0.001), though no interactions between the fungi were found (Fig. 1b).

**Fig. 2:**
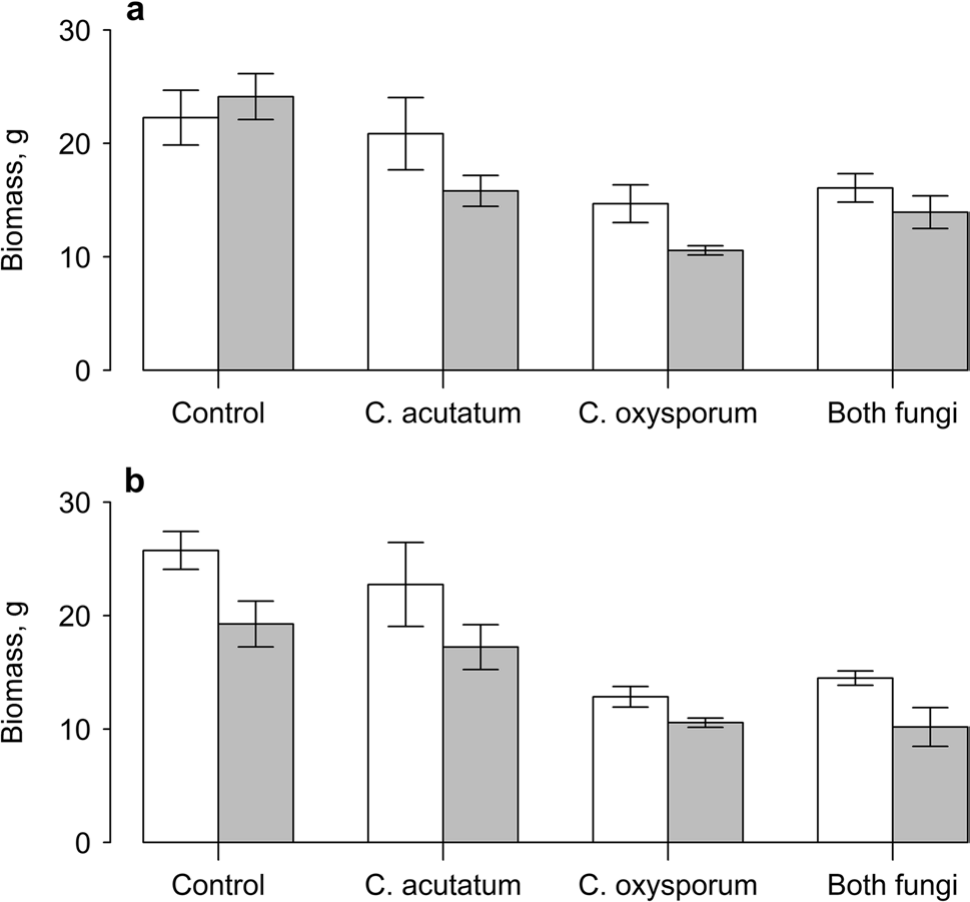
Fresh biomass of Himalayan balsam plants grown with addition of PlantWorks mycorrhizal inoculum (**a**) or Symbio mycorrhizal inoculum (**b**). White bars indicate sterilised inoculum, grey bars indicate live mycorrhizal fungi. Plants were also inoculated with no endophytes (control), *C. acutatum*, *C. oxysporum* or both endophyte fungi. Vertical bars represent ± one standard error

Overall, there was a 13% reduction in plant biomass from inoculation with PlantWorks mycorrhizal fungi, showing a weak statistical difference (*P* = 0.057). However, the reduction in height caused by inoculation with *C. oxysporum* also translated into a large reduction in biomass (main effect: *F*_1,32_ = 27.08, *P* < 0.001, Fig. 2a). In this case, there was a significant interaction between the two endophytes (*F*_1,32_ = 8.32, *P* < 0.01); if *C. acutatum* was not inoculated, *C. oxysporum* reduced biomass by 46%, but when *C. acutatum* was also inoculated, the reduction by *C. oxysporum* was only 18% (Fig. 2a). Meanwhile, addition of the Symbio inoculum significantly reduced fresh biomass, irrespective of whether the endophytes were also inoculated (main effect: *F*_1,32_ = 10.89, *P* < 0.01, Fig. 2b). Inoculation of *C. oxysporum* again reduced plant biomass by 43% overall (main effect: *F*_1,32_ = 39.33, *P* < 0.001), with the smallest plants being those inoculated with this endophyte and the mycorrhizal fungi (Fig. 2b, Tables 1 and 2).

### Insect Abundance

**Fig. 3:**
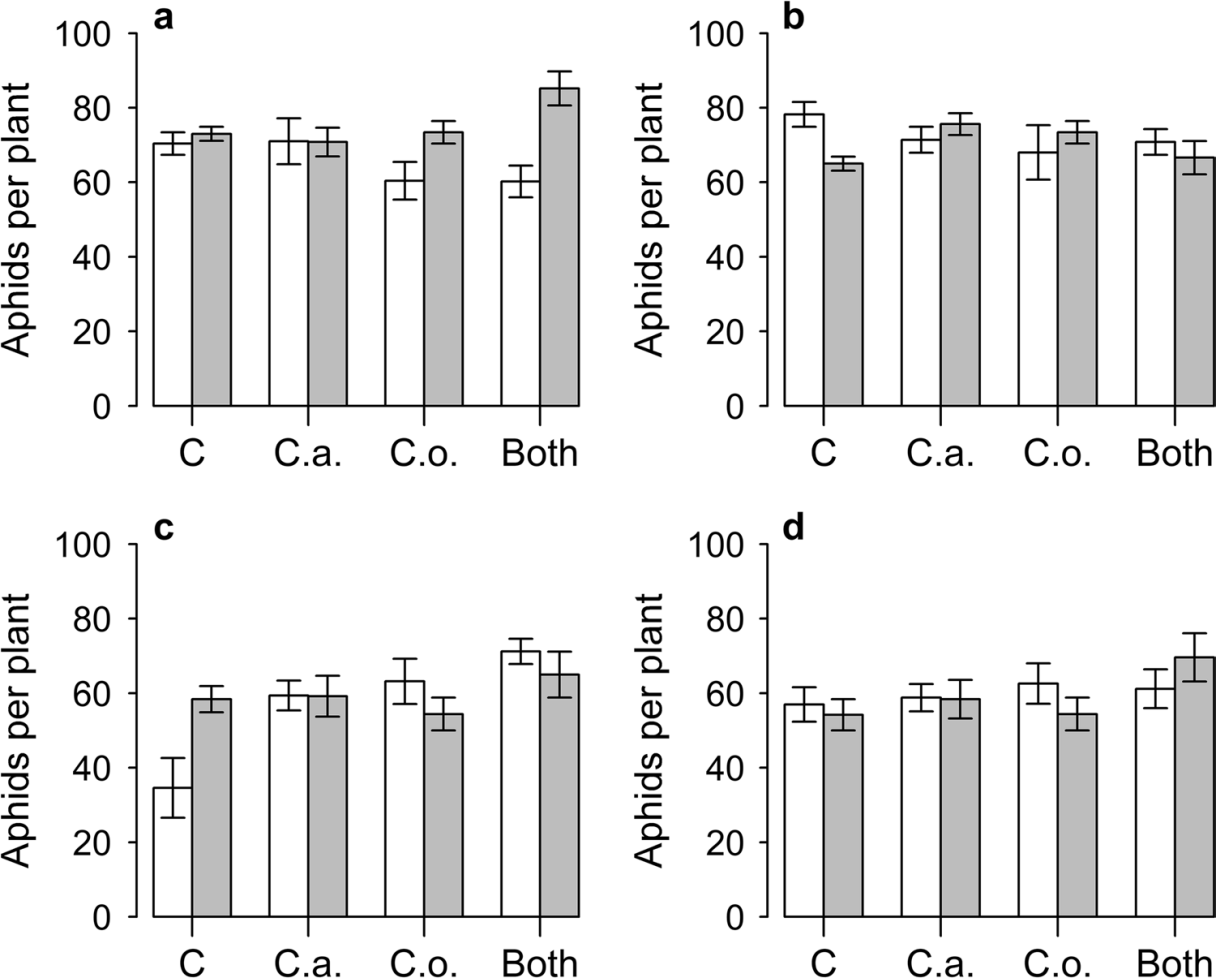
Total numbers of *Aphis fabae* on plants with (grey bars) and without (white bars) live mycorrhizal fungi addition of PlantWorks inoculum (**a**) and Symbio inoculum (**b**) and of *Myzus ornatus* with PlantWorks (**c**) and Symbio (**d**) inocula. Plants were also inoculated with no endophytes (C), *C. acutatum* (C.a.), *C. oxysporum* (C.o.) or both endophyte fungi. Vertical bars represent ± one standard error

Overall, addition of PlantWorks inoculum resulted in higher numbers of *A. fabae* per plant (main effect: *z* = 11.8, df = 1, *P* < 0.01), but this only occurred when *C. oxysporum* was also inoculated, leading to a significant interaction term between AMF and this endophyte (*z* = 9.4, df = 1, *P* < 0.01, Fig. 3a). Highest aphid numbers were found on plants inoculated with mycorrhizal fungi and both endophytes (Fig. 3a). Addition of Symbio inoculum had no effect overall, but appeared to reduce *A. fabae* numbers if no endophyte was applied, leading to a significant interaction term between the AMF, *C. acutatum* and *C. oxysporum* (*z* = 5.6, df = 1, *P* < 0.05, Fig. 3b). The response of *M. ornatus* was quite different; addition of PlantWorks inoculum only increased aphid numbers if no endophyte was present, and there was no effect of AMF overall. Meanwhile, inoculation with both *C. acutatum* and *C. oxysporum* increased the numbers of aphids per plant (main effects: *z* = 8.5, and z = 7.8 respectively, df = 1, *P* < 0.01, Fig. 3c). There was also an interaction between AMF and *C. oxysporum*, as the increase in aphids after inoculation with this endophyte only occurred when the mycorrhiza was absent (*z* = 6.5, df = 1, *P* < 0.05, Fig. 3c). A similar trend was seen with *C. acutatum,* but the interaction term was not significant. Meanwhile, with Symbio inoculum, no effect of mycorrhizal or endophyte inoculation was found, and there were no interactions between the fungi (Fig. 3d, Tables 1 and 2).

### Fungal Colonization and Infection Levels

**Fig. 4:**
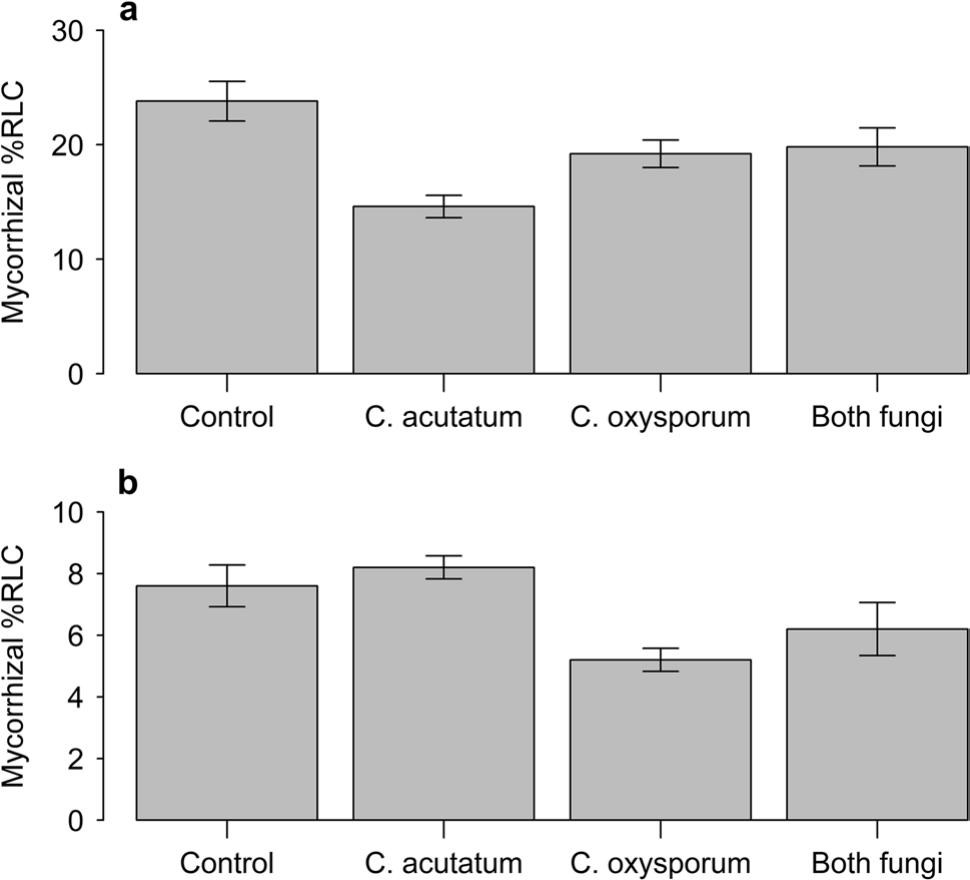
Percent root length colonised by arbuscular mycorrhizal fungi with the addition of live PlantWorks inoculum (**a**) or Symbio inoculum (**b**). Plants were also inoculated with no endophytes (control), *C. acutatum*, *C. oxysporum* or both endophyte fungi. Vertical bars represent ± one standard error

No mycorrhizal colonisation was found in any of the PlantWorks or Symbio inoculum control plants. Infection of plants with *C. acutatum* significantly reduced the level of root colonisation by PlantWorks inoculum (main effect: *F*_1,16_ = 18.13, *P* < 0.001, Fig. 4a); however, this only occurred when *C. oxysporum* was not inoculated, leading to a strong interaction between the endophytes (*F*_1,16_ = 23.54, *P* < 0.001). Overall, colonisation levels resulting from addition of the Symbio inoculum were much lower (Fig. 4b) and these were reduced by inoculation with *C. oxysporum* (main effect: *F*_1,16_ = 13.08, *P* < 0.01), with no interactions between the endophytes.

**Fig. 5:**
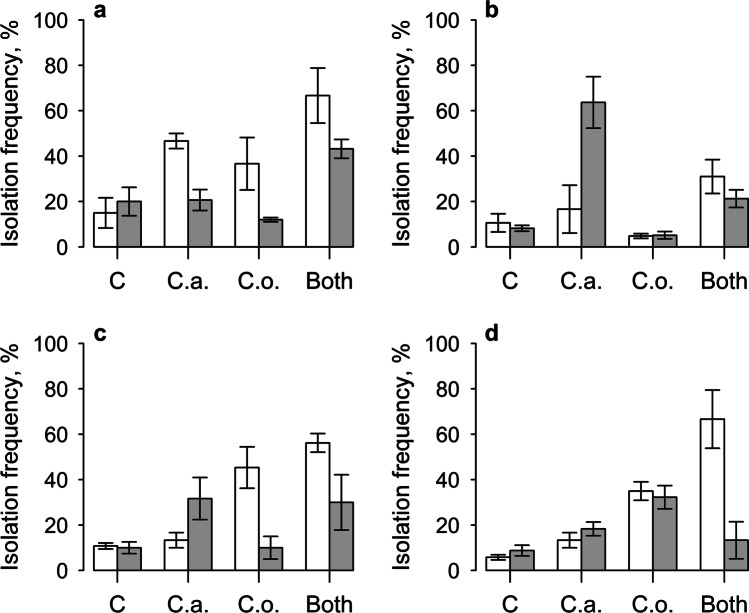
Isolation frequency of *C. acutatum* on plants inoculated with (grey bars) and without (white bars) live mycorrhizal fungi addition of PlantWorks inoculum (**a**) and Symbio inoculum (**b**) and of *C. oxysporum* with PlantWorks (**c**) and Symbio (**d**) inocula. Plants were also inoculated with no endophytes (C), *C. acutatum* (C.a.), *C. oxysporum* (C.o.) or both endophyte fungi. Vertical bars represent ± one standard error

Some background levels of infection by *C. acutatum* and *C. oxysporum* were found in the control plants, but importantly, the isolation frequencies of both endophytes were significantly higher in plants to which the respective fungi were applied (Fig. 5). Addition of PlantWorks inoculum halved the isolation frequency of *C. acutatum* from an overall value of 43% when AMF were absent, down to 21% when they were present (main effect: *χ*^2^ = 4.6, *P* < 0.05, Fig. 5a). Inoculation with *C. oxysporum* tended to increase the isolation frequency of *C. acutatum* from an overall value of 25% up to 39%, but this effect was weak (main effect: *χ*^2^ = 3.05, *P* = 0.09, Fig. 5a). Levels of infection by *C. acutatum* were lower with the Symbio inoculum and the reverse effect of mycorrhizal addition was seen, wherein the addition of AMF tended to increase infection of *C. acutatum* (Fig. 5b). However, inoculation of *C. acutatum* resulted in much higher levels of infection by this endophyte when mycorrhizas were present, compared to when they were absent, leading to a significant interaction term (*χ*^2^ = 4.5, *P* < 0.05, Fig. 5b). A further interaction was found between AMF and *C. oxysporum*, as the highest isolation frequency of *C. acutatum* was found when mycorrhizal fungi were added, but *C. oxysporum* was not (*χ*^2^ = 8.9, *P* < 0.01, Fig. 5b).

In contrast to *C. acutatum*, addition of the PlantWorks inoculum had no overall effect on infection levels of *C. oxysporum*; however, inoculation with this endophyte only increased its isolation frequency when AMF were absent, showing a significant interaction (*χ*^2^ = 6.1, *P* < 0.05, Fig. 5c). A similar effect was seen with the Symbio inoculum; inoculation with *C. oxysporum* increased infection levels of this endophyte by 41% overall when AMF were absent, but by only 9% when AMF were added (*χ*^2^ = 4.5, *P* < 0.05, Fig. 5d, Tables 1 and 2).

Ten other species of background endophytes were found in total across the experiment, these were *Acremonium strictum*, *Alternaria tenuissima*, *Chaetomium cochliodes*, *Cladosporium cladosporioides*, *Cl. sphaerospermum*, *Clonostachys rosea*, *Geniculosporium* spp., *Lecanicillium* spp., *Peniophora* spp. and *Trichoderma viride.* The *Lecanicillium* spp. and *Peniophora* spp. were sterile in culture and were identified molecularly, with GenBank accession numbers MH428682 and MH428683 respectively. In general, most plants were infected by a small number of species, with an overall mean of 2.12 ± 0.15 per plant. Addition of PlantWorks inoculum had no effect on endophyte species richness, but inoculation with *C. oxysporum* reduced species number per plant from an average of 2.6 ± 0.19 when this species was not inoculated to 1.65 ± 0.18 when it was (main effect: *z* = 2.39, *P* < 0.01). Exactly the same effect was found when *C. oxysporum* was inoculated with Symbio AMF; here the endophyte reduced species number per plant from 2.15 ± 0.18 down to 1.5 ± 0.25 (main effect: *z* = 2.1, *P* < 0.05), while the mycorrhizal fungi had no effect on endophyte species richness.

## Discussion

This is the first study of the interactions between arbuscular mycorrhizal fungi, foliar endophytes, insects and their shared host plant. We found a complex series of interactions, the outcome of which was determined by the identity of the fungi and the insects. Perhaps surprisingly, the majority of the interactions were between the spatially separated AMF and endophytes, suggesting that the mechanism is likely to be one of chemical changes in the host plant, produced and/or induced by the fungi [45].

Our first hypothesis, that adding AMF would reduce plant growth, was partially upheld, but there was a striking difference between the two inocula. Although colonisation levels resulting from addition of the Symbio inoculum were lower, the reduction in plant biomass was much greater than that resulting from addition of the PlantWorks inoculum. In neither case were the colonisation levels as high as those in natural field populations [31], so the fact that size reductions were found at relatively low colonisation levels is perhaps encouraging for the potential use of the fungi in the biological control of this weed. It is also encouraging in that this result seems to be reproducible; in a previous study, fresh biomass of *I. glandulifera* was halved when natural mycorrhizal colonisation was doubled [32]. Of the eight explanations for AMF-induced growth depression in plants listed by Jin et al. [25], that of unbalanced carbon-for-nutrient trade seems the most likely here. For an annual plant, *I. glandulifera* shows a phenomenal growth rate in the invaded range, growing up to 4 m tall, suggesting that it has a remarkably efficient root system that is highly effective at extracting soil nutrients [29, 30, 37]. Thus, it probably derives little benefit from nutrients donated by the mycorrhizal fungi, while still transferring carbon to its fungal associates. Furthermore, these results suggest that there is a high degree of ecological specificity in the association of this plant with mycorrhizal fungi [23], with the identity of the fungal species being critical in affecting plant growth. This is in line with previous studies, where differences in the performance of different inocula on the same plant have been attributed to differences in their species composition [46]. The Symbio inoculum contains more species than PlantWorks and only two (*F. mosseae* and *R. irregularis*) are common to both. If one wanted to single out particular species for in-depth study of their antagonism towards *I. glandulifera*, then extensive molecular analyses would need to be done, to determine how many of the species in the inocula actually colonised the roots, something which is very rarely done [47].

Of course, the whole point of mycorrhizal inocula is to achieve beneficial growth effects in a range of horticultural and agricultural crops and there has been some debate as to whether single or multiple species inocula are best to achieve this [48, 49]. One must also consider the economics of inoculum production; the more species in a mixture, the more likely it is that colonisation will occur, and growth benefits will be seen in a range of crops, making the product more successful [50]. Nevertheless, these results suggest that the use of Symbio inoculum in the field would be worth trialling, as it may reduce *I. glandulifera* growth, while at the same time increasing growth of native species that are more dependent on the mycorrhizal associations which are depleted by *I. glandulifera* [30, 32]. Such an approach could be a novel use of AMF in habitat restoration and could be appealing to inoculum producers [30].

More surprisingly, inoculation of plants with the endophyte *C. oxysporum* reduced both plant height and biomass with the smallest plants being produced when this endophyte and either mycorrhizal inoculum was added. There was no visual evidence of pathogenicity, with no production of leaf lesions when this species or *C. acutatum* was inoculated, similar to previous experiments with this plant [34]. This effect occurred despite this endophyte also reducing mycorrhizal colonisation by both inocula, further suggesting that the identity of fungi in the plant is more important than the amount of mycorrhizal colonisation in the roots. The performance of mycorrhizal inoculants in field conditions is highly variable and is known to be influenced by the nature and properties of soils, other soil organisms, environmental conditions and the timing of inoculation, in addition to the plant and fungal specificity effects described above [51]. Our results suggest that the identity of endophytes within the leaves of plants can also affect the functioning of the mycorrhizal fungi. While the mechanism of interaction between the spatially separated AMF and foliar endophytes is unknown, it is most likely one of metabolite production [52].

Changes in plant chemistry as a result of mycorrhizal colonisation are often detrimental to chewing insects, though sucking insects often benefit from mycorrhizal presence, through improved nutrition [26, 27]. Our second hypothesis was again only partially upheld, in that addition of PlantWorks inoculum led to an increase in numbers of *A. fabae* (but only when *C. oxysporum* was also inoculated) and had no effect on *M. ornatus.* Meanwhile, addition of Symbio inoculum had no effect on either aphid. The latter result is most encouraging, as it means that use of this inoculum in the field is unlikely to increase the reservoirs of these two crop pests. Mycorrhizal fungi appear to increase the size of the vascular bundle, thereby making aphid feeding more efficient, and leading to increases in growth and fecundity [27]. It is possible that such physiological changes do not occur in this plant or that both aphids are highly proficient in phloem location, enabling them to be unusually polyphagous. It is known that effects of AMF on aphids are most apparent at low soil nutrient levels [53], but our aim in this study was to use a standardised soil that was most similar to the nutrient-rich environments in which *I. glandulifera* grows, such as riverbanks and woodland edges [37]. Contrary to expectation, there were notable effects of endophyte inoculation, with both *C. acutatum* and *C. oxysporum* increasing aphid numbers. Our third hypothesis, that endophytes would mitigate any positive mycorrhizal effects, was certainly not upheld. If anything, endophytes tended to act with the mycorrhizal inocula to increase aphid numbers, not reduce them. It is unclear why the endophytes increased numbers of both aphids, when most previous studies have found negative effects of endophyte presence, thought to be caused by induced metabolites being carried in the phloem food supply of the aphids [17]. This is clearly an avenue for further study.

However, close inspection of the aphid data may reveal more subtle effects of the constitution of the mycorrhizal inoculum. One would expect similar effects of the endophytes on the aphids in the absence of either live mycorrhizal inoculum; however, this was not the case. For *M. ornatus* in particular, inoculation with either endophyte increased aphid numbers on plants receiving sterilised PlantWorks inoculum, but this was not the case with the Symbio inoculum. The only differences between these treatments were the composition of the non-mycorrhizal microbial community added to each and the nature of the carrier material. Both are equally likely [54] but show that our use of two controls was appropriate and that one needs to be very careful when interpreting the results of mycorrhizal addition experiments using different inoculants.

Our third hypothesis was that there would be significant interactions between the endophytes themselves and again, there was little evidence from the inoculations to support this suggestion (though see below regarding the background endophytes). In fact, the majority of significant interactions found were between the endophytes and the mycorrhizal fungi, some of which were quite complex. Addition of PlantWorks inoculum considerably reduced the infection levels of *C. acutatum* when this endophyte was inoculated, but addition of Symbio inoculum tended to have the opposite effect. Similarly, when plants were inoculated with *C. oxysporum*, isolation frequencies showed the biggest increases when the AMF were absent. A frequent criticism of endophyte inoculation experiments has been the failure to recover the fungi that were inoculated, even though significant effects of inoculation were found on insects or plant growth [17, 55, 56]. Our data suggest that mycorrhizal fungi play an important role in endophyte colonisation of their hosts, and need to be controlled for, particularly in field experiments.

AMF are known to alter the structure of foliar endophyte communities in forbs (though not necessarily the species richness of fungi) [57]. In the present study, no effect of mycorrhizal colonisation on the background community of endophytes was seen. This assemblage, as in previous work, was quite species-poor, compared with other forbs [13, 19, 32, 34]. However, there was a consistent effect of *C. oxysporum* in reducing endophyte species richness, thus suggesting that there were some antagonistic effects between endophytes, as we had hypothesised. In a previous meta-analysis involving endophytes and insects, 98% of studies did not consider the consequences of endophyte inoculation on the background community of fungi [17], yet the interactions with and effects on this community could be just as important as the inoculated fungi themselves [58]. These interactions are likely to involve the production of anti-fungal and anti-insecticidal metabolites by the various endophytes, which could have dramatic effects on the plant and associated insects [59, 60].

There is much scope for the use of arbuscular mycorrhizal fungi and endophytes in biological control programmes of weeds, either through direct effects on the weed, or indirect effects through the manipulation of plant competition or insect herbivores [28, 61, 62]. Many endophytes, including the two species used here, are pathogens of other plants and the schizotrophic lifestyle seems to be common amongst endophytes in forbs [13, 14]. As long as appropriate species of mycorrhizal fungi and strains of endophytes (non-pathogenic to crops) are selected, so that weeds do not become reservoirs of insect pests or crop pathogens, then this represents a promising avenue for future biological control programmes. However, many more multitrophic experiments of the type reported here will be needed, to unravel the complex interactions that exist between the different parts of the plant microbiome.

## Data Availability

The datasets generated during and/or analysed during the current study are available from the corresponding author on reasonable request.
